# A Bioluminescence-Based Serum Bactericidal Assay to Detect Bactericidal Antibodies Against *Neisseria meningitidis* in Human Sera

**DOI:** 10.3390/microorganisms13030595

**Published:** 2025-03-04

**Authors:** Giulia Fantoni, Ala-Eddine Deghmane, François Caron, Muhamed-Kheir Taha

**Affiliations:** 1 Institut Pasteur, Invasive Bacterial Infections, Université Paris Cité, F-75006 Paris, France; giulia.fantoni@vismederi.com (G.F.); ala-eddine.deghmane@pasteur.fr (A.-E.D.); 2 Department of Biotechnology, Chemistry and Pharmacy, University of Siena, 53100 Siena, Italy; 3 Department of Infectious Diseases, INSERM, University of Normandie Caen-Rouen, Normandie University, DYNAMICURE UMR 1311, CHU Rouen, F-76000 Rouen, France; francois.caron@chu-rouen.fr

**Keywords:** bioluminescence, bactericidal antibodies, *Neisseria meningitidis*, serum bactericidal assay, complement

## Abstract

Serum bactericidal assay (SBA) is a functional assay that evaluates infection- and vaccine-induced neutralizing antibodies representing the serological correlate of protection against *Neisseria meningitidis*. However, it is time consuming due to its readout using the enumeration of colony-forming units (CFUs), making this conventional SBA (C-SBA) difficult for large-scale use. We developed a new SBA method that takes advantage of a bioluminescence *N. meningitidis* serogroup B (BioLux-SBA). The assay development steps involved the human complement source validation, the setup of the optimal incubation time, and the assessment of intra-day and inter-day variability. BioLux-SBA was then compared to C-SBA using a serum collection of Norman children vaccinated in 2011 with MenBvac, an OMV meningococcal vaccine. While a conventional approach requests 48 h of work to test 24 sera per day, BioLux-SBA takes only 5 h to test 96 sera per day. The SBA titers (n = 10) correlated with R^2^ of 0.98 (*p*-value < 0.0001). The deposition of terminal complement components (C5b-C9) measured by flow cytometry on the bacterial surface well correlated with BioLux SBA titers. This high-throughput method to evaluate the immunogenicity of meningococcal vaccines appears to be a reliable method for an OMV meningococcal B vaccine and requires further assessment in other laboratories and against other meningococcal vaccines.

## 1. Introduction

**Figure 1:**
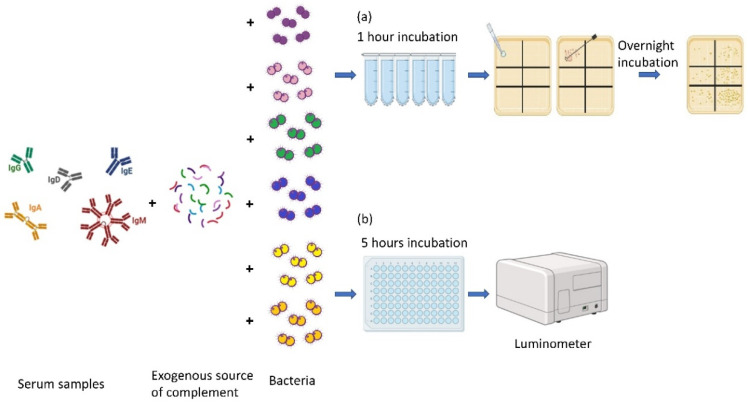
Graphical representation of the main steps of the (**a**) C-SBA and (**b**) BioLux-SBA. The serially diluted sera are incubated with an exogenous source of complement and bacteria. (**a**) In the case of the C-SBA, after one hour of incubation, the reaction mix is plated on agar plates, incubated overnight, and the day after the number of colonies is enumerated by counting. (**b**) In the case of BioLux-SBA, after five hours of incubation of a higher number of sera, the bioluminescence emitted by live bacteria is detected immediately with no additional reagents or plating by the luminometer. This figure was created using BioRender, a publicly available online software for creating scientific figures (www.biorender.com accessed on 30 August 2024).

*Neisseria meningitidis* is a Gram-negative capsulated diplococcus that can cause septicemia and meningitis in susceptible individuals, mainly in infants under 1 year of age, teenagers, and young adults [1]. There are twelve meningococcal serogroups each with a chemically distinct capsular polysaccharide; of these, six serogroups, A, B, C, W, X, and Y, cause disease [1]. Vaccination can effectively control and prevent invasive meningococcal disease (IMD) [2,3]. Capsular glycoconjugate vaccines are available for five serogroups (A, C, W, X, and Y) while the isolates of serogroup B, due to its poorly immunogenic capsule, required other vaccine approaches, like reverse vaccinology and vaccines based on outer membrane vesicles (OMVs) [1]. In 1922, Heist et al. were the first to document the presence of serum bactericidal activity in patients affected by IMD [4]. Later, Goldschneider et al. demonstrated the predictive value of SBA as a surrogate marker for the clinical efficacy of vaccines against *N. meningitidis*, tests using human complement (hSBA) being considered the current gold standard for immunogenicity assessment [5]. Other complements such as the baby rabbit complement have been employed for MenACWY vaccines, but this is inappropriate for the MenB SBA assay [6]. The SBA assay not only detects but also quantifies the titer of functional bactericidal antibodies in the serum sample of subjects who have received an anti-meningococcal vaccine. Currently, the conventional assay (C-SBA) involves the use of decomplemented serially diluted sera, the bacteria, and an exogenous source of complement devoid of intrinsic bactericidal activity [7] (Figure 1). The mixture is usually incubated for 1 h and then plated on a solid agar plate. The presence of functional antibodies in the sera leads to the activation of the antibody–complement-dependent killing; consequently, the number of colony-forming units (CFUs) in the agar plate is inversely proportional to the concentration of functional antibodies. The functional antibodies can mediate pathways leading to pathogen elimination, like receptor-mediated phagocytosis, cytotoxicity, the release of inflammatory mediators, the transport through the mucosa, and complement activation. The complement system involves enzyme cascades triggered by three pathways: the classical, the alternative, and the mannan-binding lectin [8]. In particular, the antibodies binding to the bacterial surface can activate the classical pathway. In general, these three different pathways lead to the formation of C3 convertases, which cleave C3 in the bioactive opsonin C3b and C3a, which leads to the rapid formation of the C5 convertase, which cleaves C5 into C5a and C5b. The Membrane Attack Complex (MAC) formation requires the sequential and irreversible association of C5b with the complement components C6, C7, C8 (C8β and C8αγ), and C9 [9]. The MAC (C5b-C9) disrupts the cell membrane of bacteria and forms transmembrane channels, leading to bacterial lysis [10,11]. Therefore, the activation of the complement led to the opsonization of the bacterial surface by C3b, promoting phagocytosis, and also induced bacterial lysis by the MAC assembly [11,12]. In contrast to measuring the serum antibody level by ELISA assay, the hSBA can provide valuable information on antibody-mediated functional activity, particularly their ability to mediate bacterial killing through MAC formation. However, it has several disadvantages due to its readout based on colony counting. Indeed, the CFU readout is laborious, time consuming (requires an overnight incubation step), relies on large quantities of agar plates, and is influenced by inter-operator variability [13]. For these reasons, quickly applying hSBA to large sera samples is difficult. Moreover, exogenous sources of complement with no intrinsic anti-meningococcal bactericidal activities are increasingly rare due to the wide implementation of anti-meningococcal vaccines that prompted the development of hSBA assays using the endogenous complement (enc-hSBA) [14]. The use of commercial IgG-/IgM-depleted human serum as a complement source may permit to overcome this difficulty [15,16,17]. However, this does not fix the time-consuming and laborious aspects of the current hSBA.

In case of the emergence of a new epidemic meningococcal strain or IMD outbreaks, it is crucial to rapidly obtain data on vaccine immunogenicity against the strain on a large cohort of subjects (due to inter-individual variations) to adapt the vaccination strategy if necessary [18]. However, prompt results are compromised by the conventional SBA approach.

Thus, we aimed to develop and validate a higher throughput method for screening bactericidal activity in sera using bioluminescent meningococci. This method is not intended for diagnosis nor for the evaluation of disease severity or manifestations.

## 2. Materials and Methods

### 2.1. Bacteria

The *N. meningitidis* serogroup B (MC58) [19] (serogroup B of the clonal complex ST32) was transformed with the previously described recombinant plasmid pDG34 in which the *PporB*-*luxCDABE-aph3′* was flanked by the meningococcal *pilE* gene and 120 bp downstream *pilE* gene to facilitate the recombination and allelic replacement in the parental strain MC58 chromosome. The resulting bioluminescent strain MC58lux expresses the luciferase constitutively [20]. Bacteria were cultured on GC medium (Difco, ThermoFisher Scientific, Illkirch, France), supplemented with Kellog supplements [21]. The expression of the *lux* operon was previously reported to be stable in bacteria grown on plates and during experimental infection in mice, and showed a consistent correlation between bacterial counts and the bioluminescent signal [22]. Bacteria were stored in 20% glycerol-containing GC liquid medium at −80 °C until use. A fresh culture of bacteria (16 h at 37 °C, 5% CO_2_) was prepared from frozen stocks by streaking onto a GC agar plate.

### 2.2. Serum Samples

The previously described collection of 48 serum samples from children 1–5 years of age vaccinated in 2008 with the MenBvac^®^ vaccine, NIPH, Oslo, Norway using a 2 + 1 + 1 schedule (at D0 and W6, then at M8 and M36) was used to develop the BioLux SBA and for the complement deposition assay. The sera from this collection were sampled 6 weeks after the last dose, i.e., in 2011, at a time Bexsero^®^ was not available in the area [23]. Ethical approval for the use of serum samples for research studies was provided by the regional ethics committee (Comité de Protection des Personnes Nord-Ouest-1), and written informed consent was obtained from the parents or legal guardians of every participant.

### 2.3. Complement Source Validation Test

Normal human sera (NHS) from healthy unvaccinated human donors were used as a source of complement for SBA assays. From overnight GC plate-grown culture, the bioluminescent strain MC58lux was 10-fold serially diluted in a 96-well plate (from the concentration of 10^6^ CFU/mL to 10^2^ CFU/mL) and incubated with 25% of different sources of complement in Hanks balanced salt solution supplemented with calcium chloride and magnesium chloride (HBSS^++^) (Gibco, Thermo Fisher Scientific, Illkirch, France) for one hour at 37 °C, 5% CO_2_. The reaction mix in the absence of the complement was used as a reference control. After the incubation time, 10 µL from each well was plated on a GC agar plate which was then incubated overnight at 37 °C, 5% CO_2_ for the enumeration of CFU. A validated complement serum does not show higher than 15% of killing when compared to a complement-free control [24], with no difference between the MC58lux and MC58 parent strain.

### 2.4. Conventional SBA

The bioluminescence *N. meningitidis* serogroup B, MC58lux, was grown overnight in a GC agar plate with supplements and adjusted to a 10^4^ CFU/mL concentration in HBSS^++^. The human sera were decomplemented by heating for 30 min at 56 °C and then two-fold serially diluted in different tubes, starting from a dilution of 1/4 until 1/128 in a final volume of 50 µL. A mixture containing the bacterial suspension and the complement was prepared in a proportion of 1:1, and 50 µL was added to each well containing the diluted serum. The final volume in each well was 100 µL. The reaction mix in the absence of the sera represents the negative control. The reaction was incubated at 37 °C, 5% CO_2_, for 1 h. After one hour of incubation, the entire reaction mix was plated on the GC agar plate with Kellogg supplements. The plates were incubated overnight at 37 °C, 5% CO_2_. The day after, the colony counting was performed, and the titer was assigned based on the highest serum dilution that caused 50% of the killing, with respect to the negative control. The serum bactericidal activity titers corresponded to the inverse of the final serum dilution, causing 50% killing of the inoculum after 1 h of incubation. When indicated, the luminescence emitted from the bacterial GC agar plate was acquired using the ChemiDoc Imaging System (Biorad, Marne-La-Coquette, France), and Image J 1.51K software (National Institutes of Health https://imagej.net/ version 1.51K accessed on 25 July 2024) was used to quantitate the signals.

### 2.5. Bioluminescence SBA (BioLux-SBA)

MC58lux was grown overnight in a GC agar plate with supplements and adjusted to the concentration of 8 × 10^6^ CFU/mL in HBSS^++^. The human sera were decomplemented by heating for 30 min at 56 °C and then were two-fold serially diluted in a 96-well white-wall flat plate, starting from a dilution of 1/4 until 1/128 in a volume of 25 µL, then 12.5 µL of a previously validated human complement source was added to the reaction with a final concentration of 25%. Lastly, 12.5 µL of the bacterial suspension was added to the reaction. The final assay volume was 50 µL. The reaction mix in the absence of the sera represented the reference and the negative control. The plate was incubated at 37 °C, 5% CO_2_ for 5 h. After one hour of incubation, 10 µL of each reaction was plated on the GC agar plate as an additional control of the sera-killing action. After 5 h of incubation, the luminescence of the assay plate was recorded using the Centro XS LB 960 microplate reader (Berthold Technologies, Baden Württemberg, Germany), and the data were expressed as a ratio to the reference condition without serum (BioLux-SBA titer).

### 2.6. Terminal Complement Complex Deposition Assay

The bioluminescent *N. meningitidis* serogroup B was grown overnight in a GC agar plate with supplements. A suspension containing 2 × 10^6^ CFU diluted in 50 µL of HBSS^++^ was mixed with 50 µL of sera in a 96-well plate. The final assay volume was 100 µL. The reaction was incubated for 30 min at 37 °C, 5% CO_2_. After the incubation, the bacteria were washed twice (centrifugation at 4000 rpm for 10 min) using HBSS^++^ with 1%BSA and resuspended in the same volume with a solution containing the FITC-anti-human C5b-C9 antibody (clone aE11 FITC conjugated antibody (HycultBiotech, Uden, The Netherlands)) diluted at 1:50 and incubated for 30 min at room temperature (RT) in the dark. The bacteria unstained with the fluorescence represent the negative control. After the incubation, the bacteria were washed twice in HBSS^++^ with 1%BSA, resuspended with formalin solution, and incubated for 30 min at RT in the dark. After the incubation, the plate was centrifuged at 4000 rpm for 10 min and resuspended in 100 µL HBSS^++^ with 1%BSA. The plate was acquired using the CytoFlex S (Beckman Coulter, Villepinte, France) and the data were analyzed using FlowJo, v10.10.

### 2.7. Calculations

For data analysis, a 4-parameter non-linear regression was applied to raw luminescence data obtained at different dilutions tested for each serum sample. A 4-parametric curve model provides a better fit for sigmoid curves in dose–response assays than the simple linear regression. It considers the minimum value, the maximum value, the point of inflection, and the slope of the curve.

Fitting was performed by weighting the data for the inverse of luminescenceˆ2. GraphPad Prism (GraphPad Software, v6, La Jolla, CA, USA) was used for fitting and IC50 determination. IC50 corresponds to the reciprocal serum dilution necessary to obtain 50% bacterial growth inhibition (SBA titer). To evaluate the precision of SBA assays, independent experiments were performed and the mean titer value ± standard errors (SEs) were determined for each serum from the different experiments.

The repeatability and the intermediate precision of the BioLux-hSBA were evaluated to demonstrate the assay precision. Repeatability (also termed intra-day assay precision) refers to the variation of the assay results under the same operating condition, while intermediate precision (also termed inter-day assay precision) assesses the variability of the analytical procedure over different days. The same human serum was tested on 3 different days: day one in duplicate, the second day in triplicate, and the third day in 4 replicates.

To examine the correlation between serum titers determined by the C-hSBA method and by BioLux hSBA, the results were plotted against each other as a linear regression. Pearson and Spearman correlation coefficients (Rs) and *p* values were obtained using GraphPad Prism 6 software (GraphPad Software, La Jolla, CA, USA).

## 3. Results

### 3.1. Complement Source Validation Assay

**Figure 2:**
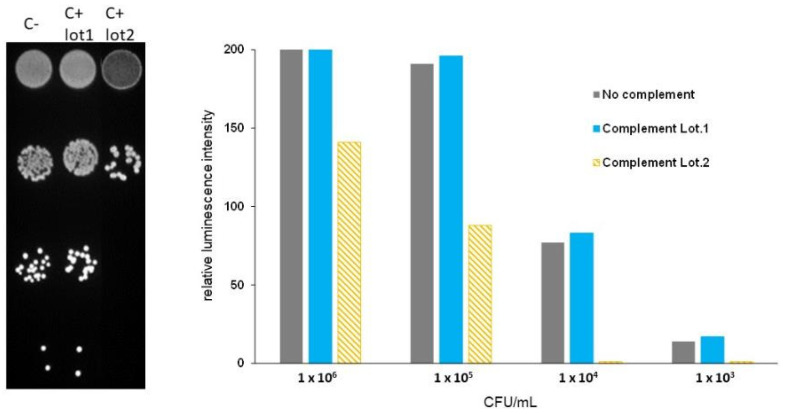
Complement source validation. Different concentrations of MC58Lux strain were tested for survival at nonimmune human serum as a source of complement (C+) at a concentration of 25% for 60 min. Bacteria without complement (C−) were used as a negative control. The luminescence emitted by the different bacterial concentrations was acquired using the ChemiDoc Imaging system (Biorad) after plating ten-fold dilution of bacteria (**left**) and the intensity of the luminescence values of the related bacterial spots was quantitated using Image J software. The results expressed as the Relative Luminometer Units (RLUs) are shown as histograms (**right**). Two lots of complement are shown: one validated (light blue) that showed no reduction in bacterial survival or bioluminescence compared to bacteria with no complement, and another non-validated (hatched orange) that showed >15% reduction in bacterial survival or bioluminescence.

The selection of a suitable source of complement represented a crucial step in setting up an SBA. To select the appropriate source of complement for measuring SBA responses, we first tested several human complements from normal human sera for their intrinsic bactericidal activity against the strain MC58lux. Bacteria incubated without complement were used as the control. The comparison between the presence and absence of a 25% complement source in the colony counting spots at different bacterial concentrations allowed the estimation of the complement-killing action. We assumed that a complement source was not bactericidal at the dilution used in the SBA (25%), when the killing did not exceed 15% of the number of target bacteria incubated without a complement source. We identified one serum out of six tested sera as a suitable complement source for the assay. Indeed, similar colony growth was observed in the presence and absence of this complement source by direct counting. Similarly, the luminescence activity emitted from bacterial spots was closely comparable for both conditions (Figure 2). This complement source was selected to carry out the subsequent tests.

### 3.2. Set Up the Incubation Time During the BioLux-SBA

**Figure 3:**
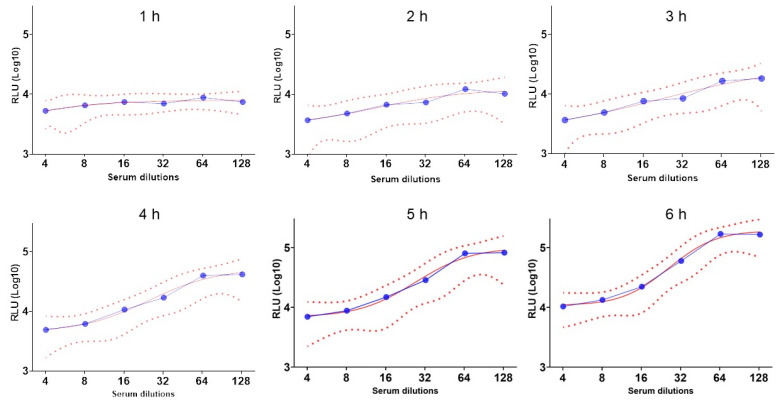
Six-hour timeline (blue curves) of a serum sample tested in BioLux-SBA where the luminescence is recorded every hour. The assay has been performed on 12 sera samples. RLUs (Y-axis) stand for Relative Luminometer Units. The solid red curves represent the fitted curves, and the dashed lines represent the confidence intervals.

In the conventional SBA, the incubation time of the reaction mix before being plated is one hour [25]. However, bacterial lysis may not immediately impact the bioluminescence signal as the luciferase enzyme and its substrate may still be available in the mixture to provide bioluminescence. In fact, after 1 h, the curve obtained was flat while the spot in the agar plate showed bactericidal activity. Therefore, we performed a time incubation setup using 12 human sera previously immunized with the MenBvac^®^ vaccine, and the bioluminescence was recorded every hour for six hours. The IC50 was compared to the CFU results of the agar plate. The results showed that the reliability of the curve (Figure 3), the IC50_,_ and the related R^2^ increased with time until reaching a plateau at 5 h, which was selected as the optimal time point to score bioluminescence.

### 3.3. Repeatability and Intermediate Precision of the BioLux-SBA

**Figure 4:**
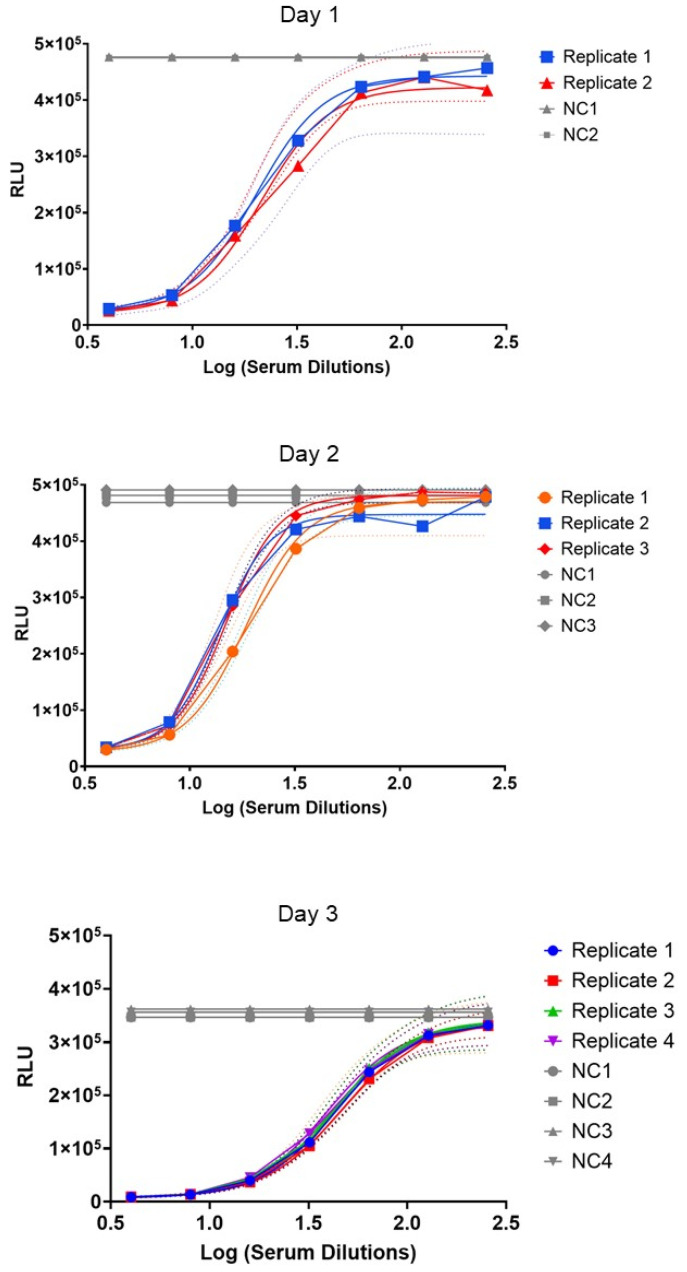
The same serum sample as in Figure 3 was tested in BioLuxSBA in duplicate on day 1, in triplicate on day 2, and in four replicates on day 3 for the evaluation of repeatability and intermediate precision. In the graph in gray (NC1, NC2, NC3, or NC4) is reported the negative control (NC) value of each replicate, which is the reaction mix (bacteria + complement) in the absence of sera. These negative controls are performed in duplicate (NC1 and NC2 on day one), in triplicate (NC1, NC2, and NC3 on the second day), and in 4 replicates (NC1, NC2, NC3, and NC4 on the third day). Samples with sera are also tested in replicates that are represented with symbols. Colored solid lines without symbols represent the curve fitting by nonlinear regression for each replicate. Dashed lines of different represent the 95% confidence interval (CI) of each replicate. RLUs (Y-axis) stand for Relative Luminometer Units.

The repeatability and the intermediate precision of the BioLux-SBA were evaluated to demonstrate the assay precision, as described in the Section 2. To evaluate the intra- and inter-day precision, the standard error (SE%) was calculated using the IC50. The repeatability showed limited variation with the SE% of 2% for the first day, 7% for the second day, and 3% for the third day (Figure 4). For the intermediate precision, the SE% was calculated using the average of IC50 from each day, with a result of 17%. These results suggest that variance is attributed to both, and repeatability and intermediate precision are not significant.

### 3.4. Correlation Between C-hSBA and BioLux-hSBA

**Figure 5:**
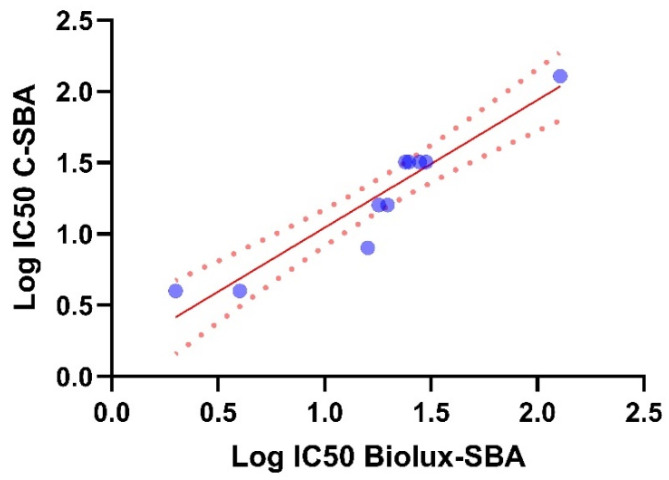
Log IC50 C-SBA vs. Log IC50 BioLux SBA (blue circle) with the red line representing the linear regression trendline, and red dotted line the 95% CI (confidence interval).

The C-SBA and the BioLux-SBA were performed in parallel for 10 sera samples to calculate the correlation between the results obtained. The results from a limited number of sera (n = 10) tested showed a good agreement between the two assays, as demonstrated by the Pearson r of 0.99 with a significant *p*-value < 0.0001 and a Spearman r of 0.96 with a *p*-value < 0.0001. The simple linear regression calculated showed an R^2^ of 0.98 with a *p*-value < 0.0001 (Figure 5). These results suggest a linear correlation between the two assays.

### 3.5. Defining the Threshold of BioLux-hSBA

The BioLux-SBA was used for the screening of bactericidal activities in 48 human sera samples of vaccinated patients that were previously described [23]. Compared to the C-hSBA established threshold, 13 of the sera (27%) showed BioLux titers lower than 4, while 35 sera (73%) showed titers equal to or higher than 4 with a complete correlation between the data of C-hSBA and BioLux-hSBA.

**Figure 6:**
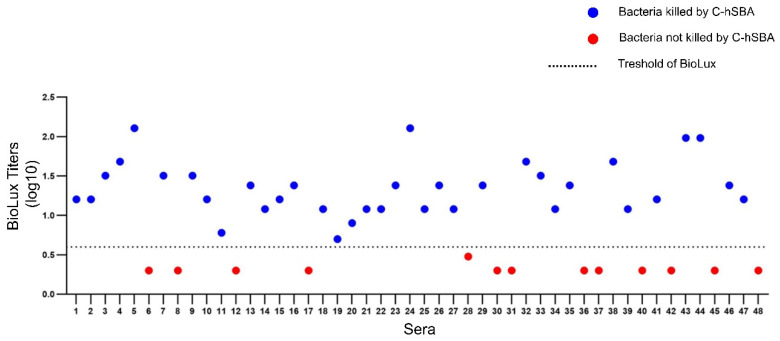
BioLux-SBA titer of 48 serum samples expressed in logarithmic scale (Y-axis) using the human complement. Red circles correspond to sera with titers of <4 (bacteria not killed) and blue circles stand for the sera with titers of = or ≥4 (killed bacteria), as determined by C-hSBA. The dashed line corresponds to the threshold of 4 of BioLux-hSBA expressed as log10 (0.6) that separates killed and not killed bacteria by C-hSBA. Number under the X-axis refers to individual sera (n = 48).

Indeed, Figure 6 shows the positive sera (C-hSBA of 4 or higher in blue circles) and negative sera (C-hSBA lower than 4 in red circles). These data suggest that the titer of 4 in BioLux-hSBA can be also used as a threshold for protective titers, as in the case of C-hSBA.

### 3.6. Terminal Complement Complex Deposition Assay

**Figure 7:**
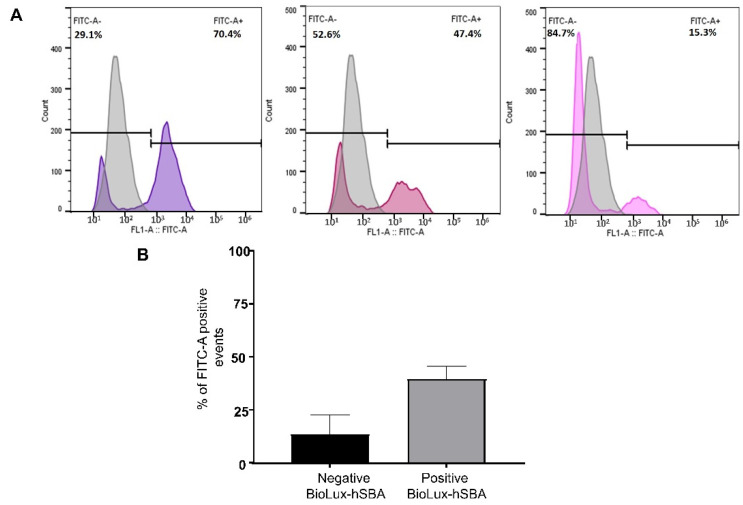
Binding of membrane attack complex on bacteria. (**A**) The histograms show the results of three sera samples (high (**left**), medium (**middle**), and low (**right**) bactericidal BioLux-hSBA titers) tested in the complement deposition assay. The gray curve stands for unstained bacteria. The colored curves correspond to bacteria stained with the FITC-anti-human C5b-C9 antibody. The percentages of positive events (bacteria with disposition of C5b-C9 complex) are indicated at the upper right corner of each graph. (**B**) Combined levels of percentage of positive events (bacteria with disposition of C5b-C9 complex) for the 48 sera tested. The black box depicts the mean and standard error of this percentage for sera with negative titer of BioLux-hSBA (titer < 4; n = 13). The gray box depicts the mean and standard error of this percentage for sera with negative titer of BioLux-hSBA (titer ≥ 4; n = 35).

We finally correlated the C-hSBA and the BioLux-hSBA titers to the levels of deposition of the C5b-C9 (the complement membrane attack complex, MAC) on the bacterial surface. The MAC deposition assay has been performed in all 48 sera samples using flow cytometry, as described in the Section 2. Three representative sera are depicted in Figure 7 with high, medium, and lower BioLux-hSBA of 128, 32, and 2 titers, respectively. As reported in Figure 7, immune sera were able to promote C5b-C9 deposition on the bacterial surface. C5b-C9 deposition levels correlated well with BioLux-hSBA titers and ranged from 70.9% (BioLux-hSBA of 128) to 47.4% (BioLux-hSBA titer of 32) and 15.3% (BioLux-hSBA titer of 2). The IC50 of 48 sera samples tested in BioLux-hSBA and the Mean Fluorescence Intensity (MFI) obtained using the C5b-C9 deposition assay showed a Spearman r correlation of 0.67, with a significant *p*-value < 0.0001 (Figure 7).

## 4. Discussion

The licensure of anti-meningococcal vaccines is not based on clinical efficacy trials but on immunogenicity assessment. The gold standard assay is the hSBA, which evaluates the presence of functional antibodies with bactericidal activity [25]. However, the conventional assay has many disadvantages, in particular concerning the colony counting readout [26]. We propose here an alternative to the conventional assay that takes advantage of the use of bioluminescent *N. meningitidis* serogroup B (BioLux-hSBA), which allows a high throughput and less laborious and time-consuming process. Such a method will impact rapid investigation during outbreaks. It can also use different sources of complement, including commercial IgG-/IgM-depleted human serum. BioLux-hSBA does not intend to replace complement sources but rather overcome the time-consuming nature of the C-hSBA.

The exogenous source of complement is one of the major biological components of the SBA assay that is difficult to standardize. Usually, the sera samples are incubated at 56 °C for 30 min to inactivate the patient-specific complement effect that is replaced by an external source, which in the case of *N. meningitidis* serogroup B, is the human complement. Bacteria can be naturally of variable susceptibility to complement-mediated killing, even in the absence of antibodies. The exogenous source of complement needs, therefore, to be tested to verify that it does not have any intrinsic killing effect on the bacteria as the bactericidal effect in SBA assay should be related only to the action of functional antibodies. The use of bioluminescent bacteria allows the unbiased evaluation of the complement-killing action by measuring the luminescence signal emitted by the bacterial spots. In this way, the operator’s bias in the evaluation and the inter-operator variability are avoided.

The incubation time is another important parameter to characterize during the development of the SBA assay. The setup of the incubation time pointed out that the luciferase activity seems to not be immediately extinct after bacterial killing. At least 5 h was required to match the bioluminescent signal to the bacterial growth on the agar plate. Then, the repeatability and the intermediate precision were assessed to evaluate the variability of the BioLux-hSBA within the same day (SE% of 2%, 7%, and 3%) and between different days (SE% of 17%). Considering that the BioLux-hSBA is a functional assay that involves several biological components like live bacteria, a human complement, and human sera samples, these variability results can be considered low.

Considering the C-hSBA as the gold standard and surrogate of protection against IMD, we performed, in parallel, the test on 10 serum samples using the conventional and the BioLux-SBA assay. We found a high correlation between the IC50 obtained with the two assays, but further validations with a larger cohort are still required. This correlation permitted the testing of 48 serum samples from vaccinated patients using the BioLux-SBA assay, and the results show that 73% of sera have a BioLux-hSBA titer higher than 4, which is the protective threshold established for the C-hSBA and is also suggested to be the threshold for BioLux-hSBA. However, further assays of comparison may still be needed using several other collections of sera.

The BioLux-hSBA is not the first high-throughput version of the SBA developed during the last years. Previous assays based on fluorescence or colorimetry attempted to develop non-plating-based readout methods. The most known is the Luminescence-SBA (L-SBA), which takes advantage of the use of a reagent able to lyse the bacteria and emit luminescence in the presence of ATP [13]. However, the reagent should be added only when the reaction mix is resuspended in PBS to avoid signal interference. Another assay is based on the addition of the metabolic indicator, alamarBlue, which is reduced during the bacterial metabolism, exhibiting both fluorescence and colorimetric change [27,28]. These assays require additional reagents to SBA and additional steps including centrifugation, the elimination of the supernatant, and the resuspension at the end of the assay. These steps need to be performed carefully to avoid technical errors. Our BioLux-hSBA does not need any of these steps because the bioluminescent signal is an end-point assay. The reaction does not need any additional reagents or steps and data can be analyzed immediately after the assay. There is no need for any special equipment, but only a luminometer microplate reader. However, the need for a luminometer and the need for further validation are limitations of the BioLux-hSBA.

In general, the SBA evaluates the capacity of the antibodies to lyse the bacteria through the activation of the complement pathways. In particular, the lysis of the bacteria is mediated by the formation of the MAC, also called the terminal complement complex (TCC), on the bacterial surface. We therefore correlated the BioLux-hSBA titers to the C5b-C9 (TCC) deposition levels by flow cytometry. This assay measures the TCC formation from the patient’s endogenous complement, targeting this complex with fluorescence antibody. However, the evaluation of MAC deposition needs to consider the potential variation between sera samples. This assay can be applied also to evaluate the level of the opsonin C3b deposition and indirectly measure the potential opsonophagocytosis of the bacteria. However, *N. meningitidis* is reported to be killed mainly by the lysis caused by the MAC pore formation on the bacterial surface [29]. The BioLux-hSBA can also be used using the endogenous complement and can therefore be an asset for enc-hSBA. It is therefore a method that can be used in both pre-clinical trials in vaccine development as well as post-implementation follow-ups.

## 5. Conclusions

The BioLux-hSBA was here a reliable approach to evaluate the immunogenicity of an OMV meningococcal B vaccine. This method is based on the use of bioluminescent strains that have been already developed in our laboratory [20]. Bioluminescence does not require adding any substrate and does not require any additional manipulation of the plates. The method seems eligible to evaluate other meningococcal groups as bioluminescent strains. However, the method requires further assessment with larger cohorts of subjects who have been immunized using other meningococcal vaccines. The improvement of hSBA methods is expected to allow faster and larger testing of sera. The improvement of hSBA methods is useful in faster testing cohorts during vaccine development and in real-time seroprevalence studies.

## Data Availability

The original contributions presented in this study are included in the article. Further inquiries can be directed to the corresponding author.
